# Individuals Appreciate Having Their Medication Record on the Web: A Survey of Attitudes to a National Pharmacy Register

**DOI:** 10.2196/jmir.1022

**Published:** 2008-11-11

**Authors:** Emelie Montelius, Bengt Åstrand, Bo Hovstadius, Göran Petersson

**Affiliations:** ^3^E-Health Institute and School of Pure and Applied Natural SciencesUniversity of KalmarKalmarSweden; ^2^Apoteket AB and School of Pure and Applied Natural SciencesUniversity of KalmarKalmarSweden; ^1^E-Health InstituteSchool of Human SciencesUniversity of KalmarKalmarSweden

**Keywords:** Medical informatics, drug information services, patient access to records, pharmacy, confidentiality, informed consent, Internet, Sweden

## Abstract

**Background:**

Many patients receive health care in different settings. Thus, a limitation of clinical care may be inaccurate medication lists, since data exchange between settings is often lacking and patients do not regularly self-report on changes in their medication. Health care professionals and patients are both interested in utilizing electronic health information. However, opinion is divided as to who should take responsibility for maintaining personal health records. In Sweden, the government has passed a law to enforce and fund a national register of dispensed medications. The register comprises all individuals with dispensed medications (6.4 million individuals, September 2006) and can be accessed by the individual online via “My dispensed medications”. The individual has the right to restrict the accessibility of the information in health care settings.

**Objective:**

The aim of the present study was to evaluate the users’ attitudes towards their access to “My dispensed medications” as part of a new interactive Internet service on prescribed medications.

**Method:**

A password-protected Web survey was conducted among a first group of users of “My dispensed medications”. Data was anonymously collected and analyzed with regard to the usefulness and design of the Web site, the respondents’ willingness to discuss their “My dispensed medications” with others, their reasons for access, and their source of information about the service.

**Results:**

During the study period (January-March, 2007), all 7860 unique site visitors were invited to answer the survey. Invitations were accepted by 2663 individuals, and 1716 responded to the online survey yielding a view rate of 21.8% (1716/7860) and a completion rate of 64.4% (1716/2663). The completeness rate for each question was in the range of 94.9% (1629/1716) to 99.5% (1707/1716). In general, the respondents’ expectations of the usefulness of “My dispensed medications” were high (total median grade 5; Inter Quartile Range [IQR] 3, on a scale 1-6). They were also positive about the design of the Web site (total median grade 5; IQR 1, on a scale 1-6). The high grades were not dependent on age or number of drugs. A majority of the respondents, 60.4% (1037/1716), had learned about “My dispensed medications” from pharmacies. 70.4% (1208/1716) of all respondents said they visited “My dispensed medications” to get control or an overview of their drugs. Getting control was a more common (*P* < .001) answer for the elderly (age 75 or above), whereas curiosity was more common (*P* < .001) for the younger age group (18-44 years).

**Conclusion:**

We found that users of the provider-based personal medication record “My dispensed medications” appreciated the access to their record. Since we found that the respondents liked the design of the Web site and perceived that the information was easy to understand, the study provided no reason for system changes. However, a need for more information about the register, and to extend its use, was recognized.

## Introduction

The development of Information and Communication Technology (ICT) is expected to have the potential to improve safety, quality, and efficiency in health care [1]. Since the first article on a computerized medical record in use almost 40 years ago [2,3], the clinical use, along with research [3], of Electronic Health Records (EHRs) has increased considerably. EHRs include longitudinal collection of health information which can be electronically accessed by authorized users. The records vary regarding the extent and kind of information displayed. Some include virtually all patient data, while others are restricted to certain types of data, such as a medication list or lab results [1,4].

Medication lists are one of the most important types of information to be included in an EHR, since they are used for filling refill requests, assessing quality, performing research, and informing computerized clinical decision support [5]. The intention of an EHR is to make important patient information available at the time and site of need. However, many patients receive health care in different settings, while most EHRs comprise information from specific settings only [1]. Thus, a limitation of clinical care may be inaccurate medication lists, since data exchange between settings is often lacking, and patients do not regularly self-report on changes in their medication [5,6].

Individuals are increasingly becoming more engaged in their health care and are interested in reading their medical records [4,7,8], and they also find it valuable to access their medical records via the Internet [9]. Individuals are expected to be important users of their own electronic health information [1]. Current and past medication information is one type of information requested by patients [4] because they regard one of the advantages of an electronic patient record (EPR) to be the ability to become better informed about their medication [10]. Though both health care professionals and patients are interested in electronic health information, opinions differ about who should take responsibility for the maintenance of the EPRs, including their accuracy, security, and accessibility.

In Sweden, the government has passed a law to enforce and fund a national register of dispensed medications. Since July 2005, all dispensed prescriptions from all pharmacies are automatically recorded in a mandatory national pharmacy register, independent of different care settings or prescribers and whether or not the individual is reimbursed for the medication. Thus, the register provides complete information on medications dispensed to the individual, with the exception of over-the-counter drugs, herbal remedies, and drugs dispensed for inpatients at hospitals [11]. The dispensed medication register is thought to suffer less from inaccuracy than a prescribed medication register, as dispensed drugs are closer to the true exposure of consumed drugs than are prescribed drugs. The record is mandatory, but the individual decides if and how he or she will use the information. The information cannot be modified by the individual. The register can be accessed by the individual online via “My dispensed medications”. We considered it important to assess a first group of users of the Web site, to suggest improvements of the service.

The aim of the present study was to evaluate the users’ attitudes towards their access to “My dispensed medications” as part of a new, interactive Internet service on prescribed medications.

## Methods

We conducted a Web survey among users of the Web-based service “My dispensed medications”. The questionnaire was developed for the purpose of this survey by two of the authors and validated by an experienced evaluator. The researchers did not have any access to the respondents’ medication records. The study was not subject to Institutional Review Board approval. All data in this study were given by the survey respondents. The respondents’ opinions were anonymously collected and analyzed with regard to the usefulness and design of the Web site, the respondents’ willingness to discuss their “My dispensed medications” with others, their reasons for access, and their source of information. The study period of 34 days ran from January 31 to March 6, 2007.

### “My dispensed medications”

**Figure 1:**
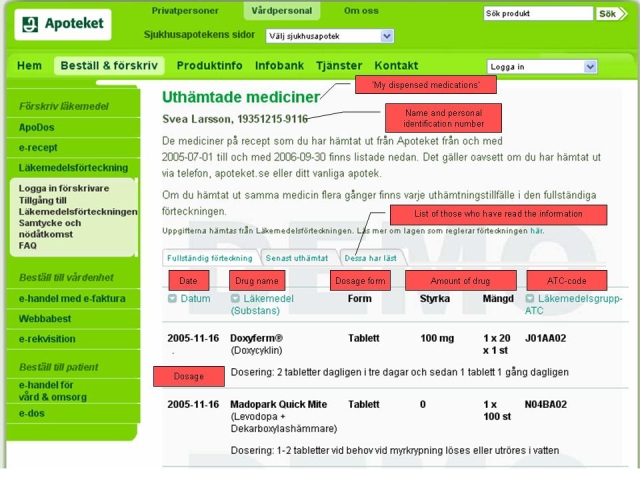
Screenshot of a demo of the web-based service “My dispensed medications” (English explanations in red boxes)

“My dispensed medications” is a Web-based service where individuals can access their list of dispensed drugs recorded in the Swedish national pharmacy register (Figure 1). Prescribers and pharmacists can only access the register with an individual’s consent, with an exception being made for physicians in case of emergency. The register is individual-based, including all dispensed prescription drugs to a person, independent of different prescribers, or whether or not the individual is reimbursed for the medication. The information is stored in the register for 15 months and thereafter cleared. In Sweden, iterations of prescriptions are filled every third month. In September 2006, 6.4 million individuals were registered in the Swedish national pharmacy register, representing 71% (6,424,487/9,047,752) of the Swedish population [11]. “My dispensed medications” is located on the Web site of Apoteket AB, The National Corporation of Swedish Pharmacies [12].

The Web survey was closed and password-protected, due to the requirement for a secure digital signature when logging on to “My dispensed medications”. The secure digital signatures are issued by Swedish banks, stored on computers, and used by the individual in combination with a personal code, and they are in general use in Sweden by several authorities and private companies for personal identification in online contacts. All Swedish citizens at the age of 18 and above can easily apply for a secure digital signature over the Internet from all major banks. In February 2007, there were about 1 million unique individuals with secure digital signatures in Sweden (personal communication, BankID, Sweden).

### Survey Design

All registered individuals using the register online to view their “My dispensed medications” during the study period were sent an invitation two seconds after they had logged on asking them to answer the survey. The registered individuals were notified that their responses would be used to improve the service “My dispensed medications” and that the survey would take less than five minutes to answer. The invitation request was not hindered by pop-up blockers. A cookie was set on the page “My dispensed medications”, preventing the invitation from being sent to the same computer twice during the study period. A unique site visitor was determined based on the use of their secure digital signature. The Web survey was not announced or advertised prior to the invitation. It was a voluntary survey. Individuals could reject the invitation or simply close the invitation screen. No incentives were offered for answering the survey.

When the visitor had approved the survey, 12 statements and 3 questions followed about their attitudes towards “My dispensed medications”, as well as 4 questions on their demographics (Tables 1, 2, and 3, and Multimedia Appendix 1). The first 7 statements were intended to answer the extent to which the respondents agreed with the purposes of the law restricting the use of the Swedish national pharmacy register [11]. The statements were displayed on two screens and the 7 questions on one screen each, yielding 11 screens in total, including the invitation screen and a final confirmation screen. By using the “back” button, respondents could review and change their answers. The respondents were allowed to skip a survey question. Checks for completeness were made after submission. All submitted surveys were analyzed. Skipped questions were reported as “no response” (Tables 1, 2, and 3).

The respondents agreed/disagreed with statements 1-12 graded on a scale ranging from 1 (do not agree at all) to 6 (fully agree) (Table 1). For the questions, the first one could only be answered with 1 of 6 alternatives, whereas questions 2 and 3 could be answered with several of the 5 alternatives (Table 2). For demographic questions, only one alternative could be chosen (Table 3). The respondents were able to provide free-text feedback to all statements and all questions on their attitudes.

### Statistics

The survey was distributed and collected with the software Easyresearch (Easyresearch Scandinavia AB, Stockholm, Sweden). Collected survey answers were analyzed using Excel (ver. 2003; Microsoft, Seattle, WA). Statistical analysis was performed using SPSS (ver. 15.0 for Windows; SPSS Inc, Chicago, IL) and Statistix 8 (Analytical Software, FL). Rate Ratio (RR) with a 95% confidence interval (95% CI) was calculated using Episheet.

The non-parametric Kruskal-Wallis test was performed to analyze the differences of sample medians and Wilcoxon rank test to analyze differences of sample medians from total median. As a measure of variability, the Inter Quartile Range (IQR) was calculated (the upper quartile – the lower quartile). χ^2^-test was used to test for association between different response alternatives and age. The rate ration [RR] with 95% CI was calculated as (negative statement _yes to question_ /negative statement _all answers_) / (positive statement _yes to question_ /positive statement _all answers_), statements graded 1-3 were considered negative and 4-6 positive. *P* < .05 was regarded significant.

## Results

During the study period 7860 unique site visitors (approximately 0.8% (7860/1,000,000) of individuals with a secure digital signature) accessed “My dispensed medications” 10,192 times. Of the 7860 unique site visitors, 2663 individuals accepted the invitation and 1716 responded to the online survey, resulting in a view rate of 0.218 (1716 survey visitors/7860 site visitors) and a completion rate of 0.644 (1716 finished survey/2663 agreed to participate) [13].

The time to answer the survey was automatically measured and lasted an average of 2.8 (median 2, IQR 2) minutes; 1% (18/1716) of the respondents submitted the survey in less than 1 minute.

The completeness rate [13] (number of responses to each question/1716 completed surveys) was between 0.995 (1707/1716) for the first screen and 0.949 (1629/1716) for the last, decreasing for every screen displayed with statements and questions on their attitudes.

### Usefulness

In general, the respondents’ opinions of the usefulness of “My dispensed medications” were high, with a total median grade of 5 (IQR 3), when asked to agree/disagree on a scale of 1 to 6 with statements on how “My dispensed medications” may be used (Table 1, “a. By means of...”). The statements “the pharmacist’s dispensing of my drugs may be safer” (*P* < .001) and “my physician may have a better decision basis for my medication” (*P* < .001) were graded above the total average. “My drug utilization may be improved” (*P* = .68) and “the information in my medical record may be improved” (*P* = .07) were in line with the total average. “I may receive better health care and treatment”(*P* < .001), “I may to a greater extent comply with my physician’s ordination” (*P* < .001), and “I may be more involved in the decisions regarding my medication” (*P* < .001) were graded under the total average. However, the differences were small, with statement medians ranging from 4 to 5.

More respondents considered “My dispensed medications” to be of greater use for the pharmacists than for the physicians (*P*< .001), when the statement “the pharmacist’s dispensing of my drugs may be safer” was compared with the statement “my physician may have a better decision basis for my medication”.

**Table 1:** Number (n) and percentage (%) of the respondents’ grading of statements (n = 1716)^*^

Survey statement		1	2	3	4	5	6	Completeness rate	No response	Grade median	IQR
**a. By means of “My dispensed medications”...**											
my physician may have a better decision basis for my medication	n	119	62	176	356	406	575		22	5	2
	%	6.9	3.6	10.3	20.7	23.7	33.5	0.987			
I may receive better health care and treatment	n	120	85	246	457	352	420		36	4	3
	%	7.0	5.0	14.3	26.6	20.5	24.5	0.979			
the information in my medical record may be improved	n	113	66	212	362	402	524		37	5	2
	%	6.6	3.8	12.4	21.1	23.4	30.5	0.978			
the pharmacist’s dispensing of my drugs may be safer	n	75	43	142	312	494	617		33	5	2
	%	4.4	2.5	8.3	18.2	28.8	36.0	0.981			
my drug utilization may be improved	n	122	83	205	379	389	510		28	5	2
	%	7.1	4.8	11.9	22.1	22.7	29.7	0.978			
I may be more involved in the decisions regarding my medication	n	152	119	288	383	313	423		38	4	3
	%	8.9	6.9	16.8	22.3	18.2	24.7	0.980			
I may to a greater extent comply with my physician’s ordination	n	162	116	262	338	335	469		34	4	3
	%	9.4	6.8	15.3	19.7	19.5	27.3	0.980			
Total median										5	3
											
**b. My opinion of “My dispensed medications” is that...**											
log on is easy	n	44	61	115	212	449	783		52	5	2
	%	2.6	3.6	6.7	12.4	26.2	45.6	0.970			
the information is easy to understand	n	18	53	132	257	523	672		61	5	2
	%	1.0	3.1	7.7	15.0	30.5	39.2	0.964			
I get a good overview of my drugs	n	23	13	42	153	417	1004		64	6	1
	%	1.3	0.8	2.4	8.9	24.3	58.5	0.963			
the information is valuable to me	n	16	24	87	211	487	820		71	5	1
	%	0.9	1.4	5.1	12.3	28.4	47.8	0.959			
the appearance of the Web page is good	n	33	45	112	304	546	611		65	5	2
	%	1.9	2.6	6.5	17.7	31.8	35.6	0.962			
Total median										5	1

^*^ The statements were graded on a scale of 1 to 6 according to extent of agreement, with grade 1 being “do not agree at all” and grade 6 being “fully agree”. IQR, Inter Quartile Range, is calculated as upper quartile – lower quartile.

### Design

Asked about their opinion on the design of the Web site “My dispensed medications” (Table 1, “b. My opinion of...”), respondents were generally positive, returning a total median grade of 5 (IQR 1). The statements “I get a good overview of my drugs” followed by “the information is valuable to me” were graded above the total median (*P* < .001 and *P* = .01 respectively). “Log on is easy” was in line with the total median (*P =* .19). “The information is easy to understand” and “the appearance of the Web page is good” were graded high, although below the total average (*P* < .001 and *P* < .001 respectively) (Table 1). The high grades for the statements (*P*-values given in the same order as the statements in Table 1 under “b. My opinion of 'My dispensed medications' is that...”) were not dependent on age (*P* = .24, *P* = .91, *P* = .55, *P* = .92, and *P* = .52 respectively) or number of drugs stated, except for the statement “the information is valuable to me” (*P* = .71, *P* = .62, *P* = .75, *P* = .03, and *P* = .12 respectively).

### Source of Information

A majority of the respondents, 60% (1037/1716), had learned about “My dispensed medications” from pharmacies (Table 2). In general, the respondents included free-text comments in the range of 3-13% (49/1716; 231/1716) for different statements and questions. Comments to at least one statement or question were submitted with 27% (464/1716) of the surveys. When the free-text comments on the respondent’s source of information were analyzed, 102 respondents could be added to the category “from pharmacies”, yielding the fact that 66% ((1037+102)/1716) of the respondents had learned about “My dispensed medications” from pharmacies. Many of these comments indicated that the visitor had learned about the service not only at local pharmacies but also at the pharmacies’ shared Web site.

### Reasons for Access

**Figure 2:**
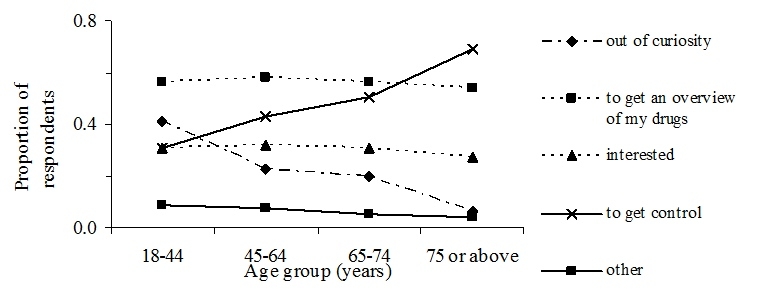
Frequency distribution of respondents’ answers to the question, “ Why did you take a look at ‘My dispensed medications’?”

The respondents visited “My dispensed medications” primarily to get an overview of their drugs and to get control, with 24% (414/1716) of the respondents acknowledging both motives and 70% (1208/1716) acknowledging either overview or control, or both, as reasons for their access. Accessing the Web site out of interest and curiosity were less common reasons, with 45% (771/1716) answering one or both (*P* < .001) (Table 2). To get control was a more common (*P* < .001) answer for the elderly (75 or above), whereas curiosity was more common (*P* < .001) for the younger age group (18-44) (Figure 2). Those who did not identify with any of the four response alternatives numbered 5% (91/1716), only answering “other”.

### Willingness to Share “My dispensed medications”

**Figure 3:**
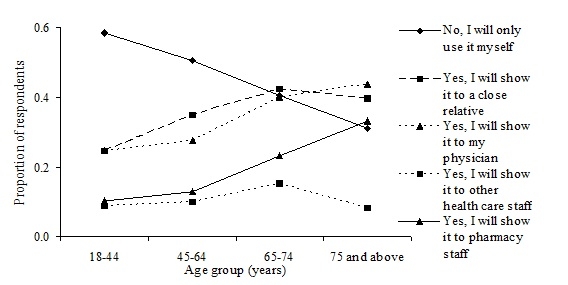
Frequency distribution of respondents’ answers to the question, “In the future, will you show or discuss your ‘My dispensed medications’ with another person?”

Respondents were keener to share their record with a close relative or their physician than with the pharmacy and other health care staff (*P* < .001) (Table 2). Respondents’ willingness to share “My dispensed medications” increased with age, except for sharing with other health care staff, which was low for all age groups (Figure 3).

Giving high grade to the statement “my physician may have a better decision basis for my medication” was well in accordance with answering yes to the question “In the future, I will show “My dispensed medications” to my physician” (RR = 0.56, 95% CI 0.44-0.71). The same relationship was found between “the pharmacist’s dispensing of my drugs may be safer” and “In the future, I will show “My dispensed medications” to the pharmacy staff” (RR = 0.48, 95% CI 0.30-0.76).

**Table 2:** Number (n) and percentage (%) of respondents for different response alternatives to 3 questions

	Total (n = 1716)		Age 18-44 (n = 614)		Age 45-64 (n = 810)		Age 65-74 (n = 177)		Age 75 or above (n = 48)		No response (n = 67)	
	n	%	n	%	n	%	n	%	n	%	n	%
How did you get to know about “My dispensed medications”?^*^												
by a physician	35	2.0	7	1.1	21	2.6	7	4.0	-	-	-	-
by a health care staff	11	0.6	2	0.3	8	1.0	-	-	-	-	1	2
by the pharmacy	1037	60.4	374	60.9	497	61.4	117	66.1	34	71	15	22
via papers/television	113	6.6	36	5.9	60	7.4	12	6.8	3	6	2	3
via a closely related	45	2.6	19	3.1	21	2.6	3	1.7	2	4	-	-
other	404	23.5	171	27.9	186	23.0	36	20.3	7	15	4	6
no response	71	4.1	5	0.8	17	2.1	2	1.1	2	4	45	67
**Why did you take a look at “My dispensed medications”?^†^**												
out of curiosity	475	27.7	251	40.9	181	22.3	35	19.8	3	6	5	8
to get an overview of my drugs	958	55.8	347	56.5	471	58.1	100	56.5	26	54	14	21
interested	521	30.4	190	30.9	260	32.1	54	30.5	13	27	4	6
to get control	664	38.7	189	30.8	347	42.8	89	50.3	33	69	6	9
other	132	7.7	54	8.8	63	7.8	9	5.1	2	4	4	6
no response	60	3.5	3	0.5	6	0.7	4	2.3	1	2	46	69
**In the future, will you show or discuss your “My dispensed medications” with another^‡^**												
I will only use it myself	862	50.2	360	58.6	409	50.5	72	40.7	15	31	5	8
Yes, I will show it to a closely related	534	31.1	152	24.8	284	35.1	75	42.4	19	40	4	6
Yes, I will show it to my physician	472	27.5	152	24.8	223	27.5	71	40.1	21	44	5	8
Yes, I will show it to other health care staff	171	10.0	55	9.0	81	10.0	27	15.3	4	8	4	6
Yes, I will show it to the pharmacy staff	232	13.5	64	10.4	105	13.0	41	23.2	16	33	6	9
no response	70	4.1	9	1.5	9	1.1	-	-	2	4	50	75

^*^Only one of the options could be chosen. Completeness rate 0.973.

^†^Several options could be chosen. Completeness rate 0.965.

^‡^Several options could be chosen. Completeness rate 0.959.

### Demographics

Demographics showed that 28% (488/1716) of the respondents resided in one of the three major Swedish cities of Stockholm, Göteborg, or Malmö, 40% (686/1716) in other cities, and 27% (469/1716) in the countryside (Table 3). The respondents seem geographically representative, since the respondents’ residences corresponded with the Swedish population as a whole with 76% (6,897,691/9,047,752) living in cities and 24% (2,150,061/9,047,752) in the countryside [14].

**Table 3:** Demographics in number (n) and percentage (%) of respondents (n = 1716)^*^

	Total		Men		Women		No response	
	n = 1716	%	n = 873	%	n = 771	%	n = 72	%
**Age^†^**								
18-44 years	614	35.8	201	32.7	410	66.8	3	0.5
45-64 years	810	47.2	494	61.0	315	38.9	1	0.1
65-74 years	177	10.3	137	77.4	39	22.0	1	0.6
75 years and above	48	2.8	40	83.3	7	14.6	1	2
no response	67	3.9	1	1.5	-	-	66	99
								
**Number of dispensed prescriptions**								
0	33	1.9	19	57.6	14	42.4	-	-
1-5	500	29.1	262	52.4	236	47.2	2	0.4
6-10	364	21.2	203	55.8	160	44.0	1	0.3
11-15	186	10.8	86	46.2	100	53.8	-	-
more than 15	509	29.7	280	55.0	227	44.6	2	0.4
no response	124	7.2	23	18.5	34	27.4	67	54
								
**Place of residence**							
Stockholm/Göteborg/Malmö	488	28.4	280	57.4	206	42.2	2	0.4
an other city	686	40.0	347	50.6	336	49.0	3	0.4
the countryside	469	27.3	242	51.6	225	48.0	2	0.4
no response	73	4.3	4	5.5	4	5.5	65	89

^*^Completeness rate 0.961 for age, 0.985 for gender, 0.949 for number of dispensed prescriptions and 0.957 for place of residence.

^†^Individuals younger than 18 years old were not eligible to participate in the study, since they are not allowed a secure digital signature and thereby not able to get online access to “My dispensed medications”.

**Figure 4:**
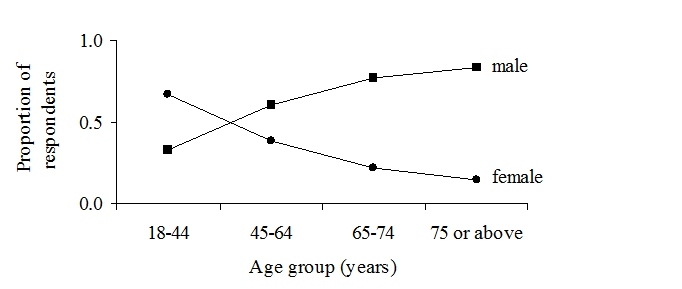
Frequency distribution of respondents’ per gender and age groups

In the age group 18-44 years, more women, 66.8% (410/614), than men responded to the survey, in contrast to the older age groups 45-64 years, 65-74 years, and 75 years and above, in which more men than women responded, at rates of 61.0% (494/810), 77.4% (137/177), and 83.3% (40/48) respectively (Figure 4).

## Discussion

We found that the first group of users of “My dispensed medications” appreciated having their medication record on the Web, and that they had a generally positive attitude towards the Web-based service. The respondents found the information valuable and easy to understand. They primarily visited the Web site to get control and see an overview of their drugs.

### Representativeness

The generalizability of Web surveys may be limited by selection bias due to the non-representative nature of the Internet population, as well as the volunteer-effect of self-selected participants [15]. The respondents in the present study comprise individuals using the Web-based service “My dispensed medications”, and thus the generalization of results to non-users should be done with care. The results of the present study are assumed to be representative for individuals who are registered in the Swedish national pharmacy register holding a secure digital signature and who have chosen to use the service “My dispensed medications”. The individuals with a secure digital signature were 18 years and older, presumably interested in ICT and health, representing the first group of users. An overestimation of positive attitudes might occur due to the nature of the early adopters [16]. However, due to restrictions in the Swedish legislation and relatively few visitors to the Web site “My dispensed medications”, we were not able to contact potential survey respondents in ways which may have generated a more representative sample. Nor could we categorize the non-respondents to assess the difference between those who chose to answer the survey and those who did not. Due to the anonymous nature of the survey, there were no means to validate the survey answers given by the respondents, by comparing them with other sources of information.

### Early Adopters

In spite of the potential value of, and positive response to, the present study, relatively few of the registered individuals in the Swedish national pharmacy register have accessed “My dispensed medications”. Although about two thirds of the respondents stated that their main source of information about the service was the pharmacies, the marketing seems to be insufficient. Also, the moderate penetration of the secure digital signature is a restriction to widespread access. Thus, we expect that only the early adopters [16] have started to use the service, about one year after its introduction on the Web.

### Usefulness

The respondents’ perception of the usefulness of “My dispensed medication” was in good accordance with the aims stated in the law (ie, to achieve a better decision basis for medications, provide the registered individual with care or treatment, supplement the individual’s health care record, assist the dispensing pharmacist, and facilitate the registered individual’s drug utilization) [11]. However, the similarly positive responses to these different statements indicated that the respondents might have had difficulties distinguishing between the different aims.

### Personal Access

The information in the national pharmacy register is available for registered individuals online via the service “My dispensed medications” and at the pharmacy counter. We found that the main reason respondents visited “My dispensed medications” was to get an overview and to get control of their drugs, followed by their interest and curiosity (Figure 2). That to get an overview and to get control were more common reasons than interest and curiosity indicates that the register is of genuine value to the respondents. For the elderly, to get control was the most common reason, whereas it was curiosity for the younger age groups. Few respondents used the response alternative “other”, indicating that the suggested response alternatives well described the respondents’ reasons for visiting “My dispensed medications”. That the service in fact gave the respondents a good overview of dispensed drugs and valuable information favors the view that patients appreciate access to their own data. Others have reported that patients want access to their EPR to be better informed about their health care and medication [10], to enhance their understanding of their medical condition, and also to facilitate their care at home [4]. For the latter, medication information, along with lab results and medical history, are most likely requested [4].

From the individual’s point of view, the safe access to his or her medication record must be easy with regard to Web site design. It seems that access to the pharmacy register is adequate, since we found that the respondents considered that logging on to “My dispensed medications” was easy. However, this might not be true for a larger population, as the Web survey was only conducted among those who had successfully logged on to the Web site. It seems that the respondents considered the Web site “My dispensed medication” to have a logical and well-structured design, since there were high grades of agreement for “the appearance of the Web page is good”, “the information is easy to understand”, and “I get a good overview of my drugs”. The high grade was not dependent on age or number of drugs, indicating that the service provided a clear overview with a high level of understandability, even for those with many drugs listed.

### Access After Conditioned Consent

There is a need to reduce overconsumption of pharmaceuticals, since excessive prescriptions might result in uncontrolled side effects and extra costs. “My dispensed medications” could be described as a provider-based personal health record [17] for dispensed medications, where the individual has the right to restrict the accessibility of the information to specific individual health care professionals, with full disclosure of those who have accessed the information. We found that the respondents were keener to share their record with a close relative or their physician than with pharmacy staff.

The finding that respondents considered “My dispensed medications” to be of great use to both pharmacists and physicians suggests that the respondents might suppose that pharmacists and physicians already have access to their medication records. However, pharmacists and physicians are dependent on the individual’s consent to view a patient’s medication record, with an exception made for physicians in the case of an emergency. Since willingness to share “My dispensed medications” increased with age, the benefits from the national pharmacy register might first be apparent for elderly persons (Figure 3). However, this might not depend on age per se, but rather an elderly person’s greater need of health care or, perhaps, a greater trust in health care professionals.

It seems reasonable that all medications should be screened at the point of care. Since the individual might visit several physicians, sometimes involving different, non-communicating EHRs, the physician might not be informed about medications prescribed by others. In Sweden, the national pharmacy register provides a complete, individually dispensed medication record, which would help when a person is visiting several physicians. Our study revealed that only about one third of the respondents were willing to show their physician their “My dispensed medications”. In the context of uncontrolled side effects with increased health care costs, one third might seem to be a small proportion; however, the same respondents agreed to a large extent with the statement that “my physician may have a better decision basis for my medication”. This indicates that the respondents who had realized that “My dispensed medications” may help their physician were also willing to share their medication record with their physician. Also, free-text comments indicated that some of the respondents had not understood that their “My dispensed medications” was not available to their physician and pharmacy staff without their consent. This implies that more information is needed about the clinical advantages for individuals when sharing their medication records with prescribers and pharmacists.

### A Nation-Wide Dispensed Medication Record

For privacy reasons, personal control over who can access the information is pivotal to deploy successfully a mandatory, nation-wide register. Individuals expect EHRs to be safe and their privacy to be respected [18], and they wish to be able to decide for themselves who else can access their record [10]. How to balance these personal confidentiality aspects with the demand for safe prescribing is a subject for continued debate. Internationally, two models for making personal health records available have been presented: the opt in model and the opt out model [5, 19-21]. Denmark and Sweden have chosen an alternative model with a legally enforced, mandatory collection of dispensed prescriptions, in combination with personal access and control. The Scandinavian approach seems to be well-balanced with public support, as there have been remarkably few public concerns raised so far.

### Internet Use and Gender Differences

The increasing use of the Internet still seems to indicate some gender differences. Internet use in Sweden is high, with a majority of the Swedish population (16-74 years of age) using the Internet. The number of men using the Internet is somewhat higher in the older age groups, relative to the number of women [14]. This is also reflected in our study in which men were the predominant respondents aged 45 years and above (Figure 4). However, the Internet use of health information has been reported to be dominated by women [22], especially young and middle-aged women [23]. This might explain the dissimilar numbers of men versus women of different age groups in our study (Figure 4). The deciding factor for the younger age group (18-44) may not be about technological experience and enthusiasm, but rather an interest in health-related issues.

### Potential Use

If used more extensively, the register might convey several advantages for users, as well as for health care generally. By using the register, the individual might have a better overview and control, helping to consume pharmaceuticals more accurately. Physicians might have better grounds for their future prescribing and pharmacists for future counseling. Whether an extended use of the register will improve drug utilization, making it more cost effective, remains to be studied. First, efforts must be made to extend the use of the register. Our study reveals that there seem to be few obstacles to the use of the register itself; rather, the limiting factor is insufficient knowledge about the register.

### Conclusion

We found that users of the provider-based personal medication record “My dispensed medications” appreciated access to their record. Keeping in mind the limitations of a Web survey, we considered it important to assess a first group of users of the Web service to be able to suggest improvements to the service. Since we found that the respondents liked the design of the Web site and perceived that the information was easy to understand, the survey provided no reason for changes. However, a need for more information about the register, to extend its use, was recognized.

## Multimedia Appendix 1

Survey questions translated into English

## Abbreviations

CI: confidence interval
EHR: electronic health record
EPR: electronic patient record
ICT: information and communication technology
IQR: inter quartile range
RR: rate ratio
