# Supplementary material for: Individuals Appreciate Having Their Medication Record on the Web: A Survey of Attitudes to a National Pharmacy Register
Source: J Med Internet Res. 2008 Nov 11;10(4):e35. doi: 10.2196/jmir.1022 (PMC2629371; doi:10.2196/jmir.1022)
Supplement: Supplementary file 1 [file jmir_v10i4e35_app1.pdf]

Survey questions translated into English

By means of 'My dispensed medications':
(Grade on a scale 1-6 according to extent of agreement; grade 1 being 'not agree at all' and grade 6 'fully agree'.)

1. my physician may have a better decision basis for my medication
2. I may receive better healthcare and treatment
3. the information in my medical record may be improved
4. the pharmacist's dispensing of my drugs may be safer
5. my drug utilization may be improved
6. I may be more involved in the decisions regarding my medication
7. I may to a greater extent comply with my physician's ordination

My opinion of 'My dispensed medications' is that:
(Grade on a scale 1-6 according to extent of agreement; grade 1 being 'not agree at all' and grade 6 'fully agree'.)

1. log on is easy
2. the information is easy to understand
3. I get a good overview of my drugs
4. the information is valuable to me
5. the appearance of the web page is good

How did you get to know about 'My dispensed medications'?

1. by a physician
2. by a healthcare staff
3. by the pharmacy
4. via papers/television
5. via a closely related
6. other

Why did you take a look at 'My dispensed medications'?

1. out of curiosity
2. to get an overview of my drugs
3. interested
4. to get control
5. other

In the future, will you show or discuss your 'My dispensed medications' with another person?

1. I will only use it myself
2. Yes, I will show it to a closely related
3. Yes, I will show it to my physician
4. Yes, I will show it to other healthcare staff
5. Yes, I will show it to the pharmacy staff

How many dispensed prescriptions are listed in your 'My dispensed medications'

1. 0
2. 1-5
3. 6-10
4. 11-15
5. more than 15

Gender
1. male
2. female

Age

1. 18-44 years
2. 45-64 years
3. 65-74 years
4. 75 years and above

Place of residence

1. Stockholm/Göteborg/Malmö
2. an other city
3. the countryside
